# Comparison of the Spine and Hip BMD Assessments Derived from Quantitative Computed Tomography

**DOI:** 10.1155/2015/675340

**Published:** 2015-07-27

**Authors:** Xiao-Hui Ma, Wei Zhang, Yan Wang, Peng Xue, Yu-Kun Li

**Affiliations:** ^1^Department of Radiology and Orthopaedic Biomechanical Laboratory of Hebei Province, The Third Hospital of Hebei Medical University, Shijiazhuang, Hebei 050000, China; ^2^Department of Endocrinology, The Third Hospital of Hebei Medical University, Shijiazhuang, Hebei 050000, China

## Abstract

Quantification of bone mineral density (BMD) is being used as the main method to diagnose osteoporosis. Dual-energy X-ray absorptiometry (DXA) is the most common tools for measuring BMD. Compared to DXA, quantitative computed tomography (QCT) can determine in three dimensions the true volumetric BMD (vBMD) at any skeletal site. In addition to the spine, the hip is an important site for axial BMD measurement. This study examines lumbar spine and hip BMD of Chinese adults by QCT. Age related changes in bone mass derived by QCT measurements were determined. The osteoporosis QCT detection rates at the spine and hip are assessed in both female and male, and agreement of skeletal status category between the spine and hip in older adults is also assessed.

## 1. Introduction

Quantification of bone mineral density (BMD) is being used as the main method to diagnose osteoporosis. Several clinical techniques for BMD measurement are available, including dual-energy X-ray absorptiometry (DXA), quantitative computed tomography (QCT), and magnetic resonance (MR) techniques. DXA is the most common tools for measuring BMD. Compared to DXA, QCT can determine in three dimensions the true volumetric BMD (vBMD) at any skeletal site. It is less affected by surrounding tissues, eliminates the posterior vertebrae elements, and facilitates analysis of trabecular and cortical bone separately [1]. QCT has been utilized for measuring spinal BMD [2, 3]. According to the International Society for Clinical Densitometry (ISCD) positions QCT of the spine can be used to predict spinal fractures, to monitor BMD changes, and to initiate treatment [4]. In addition to the spine, the hip is an important site for axial BMD measurement [5], 3D QCT systems for measurement at the hip have been proposed and developed for research. CTXA Hip (a commercial QCT BMD analysis system) uses 3D QCT volume data sets to generate bone projection images that visually look like those generated by DXA. However, CTXA Hip exploits the anatomical detail in the 3D QCT data set to segment bone from surrounding tissues rather than relying on the dual-energy imaging method of DXA. Compared to DXA, CTXA Hip may provide more information. Nevertheless, the higher radiation exposure due to a dedicated QCT exam in comparison to a DXA must be considered.

At present, according to the diagnostic criteria for DXA established by the World Health Organisation (WHO) in 1994 in the clinical diagnosis of osteoporosis, osteoporosis is diagnosed by central DXA if the *T*-score of the lumbar spine or hip is −2.5 or less. However, the WHO diagnostic classification cannot be applied to *T*-scores from measurements other than DXA at the femur neck, total femur, lumbar spine, or one-third distal (33%) radius because those *T*-scores are not equivalent to *T*-scores derived by DXA. Thus equivalence with a DXA measurement cannot be achieved to QCT of the spine [6]. For the QCT vBMD of spinal trabecular bone, thresholds of 80 mg/cm^3^ for osteoporosis QCT (equivalent to a DXA *T*-score of −2.5) were suggested by the ISCD in 2007 [4] and by the American College of Radiology [7]. The areal BMD (aBMD) measurements and *T*-scores derived from CTXA and DXA are probably close [8]. Khoo study concluded QCT aBMD appropriately adjusted can be evaluated against NHANES reference data to diagnose osteoporosis [9].

In this study, CTXA Hip BMD measurements for total hip and femoral neck were compared to QCT Pro volumetric spine BMD measures. The aim of the present study was to determine (1) age related changes in bone mass in eastern Chinese derived by QCT and (2) the osteoporosis QCT detection rates at the spine versus hip and comparison of bone mass diagnostic classification by QCT vBMD and aBMD.

## 2. Design and Methods

### 2.1. Participants

The current study included 496 females and 330 males aged 20 to 84 years. All subjects were divided into two groups both females and males: young (age, <50 years), and older (age, ≥50 years). Study exclusion criteria included a history of renal failure, alcoholism, chronic colitis, leukemia, multiple myeloma, rheumatoid arthritis, metabolic and endocrine diseases, or bone tumors. Likewise, none of the participants were taking any medications that were likely to affect bone or soft tissue metabolism, such as glucocorticoids. The study protocol and procedures were approved by the ethics committee of the hospital. All of the participants provided written informed consent before any measurements were obtained.

### 2.2. QCT Measurements of BMD

All subjects were scanned using CT (*Somatom Sensation 16*, Siemens, Erlangen, Germany). Scan was performed using the following parameters: 120 Kv, 125 mAs, 1 mm slice thickness, and 500 Mm field of view (FOV).

QCT studies were performed using the QCT Pro calibration phantom and software system with the CTXA Hip analysis module (Mindways Software, Inc., Austin, TX). For lumbar spine trabecular BMD measurement, vertebrae from L2 to L4 were scanned in the supine position. Elliptical regions of interest were put in the midplane of three vertebral bodies (L2–L4) in the trabecular bone automatically. In CTXA Hip area BMD measurements, an anterior-posterior computed radiograph was obtained by the scanner from the iliac crest to mid-thigh, and the top of the femoral head to approximately 1 cm below the inferior extent of the lesser trochanter was defined graphically to define the scanning region.

### 2.3. The Diagnostic Criteria for Osteoporosis QCT

Based on these guidelines [6, 7], volumetric trabecular BMD values from 120 to 80 mg/cm^3^ are defined as osteopenic QCT and BMD values below 80 mg/cm^3^ as osteoporosis QCT. CTXA Hip BMD estimates provide the same clinical utility as that afforded by DXA. Osteoporosis is diagnosed by central DXA if the *T*-score of the lumbar spine or hip is −2.5 or less. Low bone mass or osteopenia is classified as a *T*-score of −1.0 to −2.5. According to WHO definition, these criteria should be restricted to postmenopausal females and aged males. In order to unify expression, all subjects were classified hereby in this study.

### 2.4. Statistical Analysis

All data analysis was performed using SPSS13.0; the incidence rates of osteoporosis QCT detection were calculated by QCT BMD measurements in males and females, the comparison of rates was conducted using chi-square test. The correlations between vBMD and aBMD variables were investigated using the Pearson correlation test for normally distributed variables or Spearman correlation for nonnormally distributed variables. The consistency was checked using Kappa-test. All statistical tests were two-tailed, and *P* < 0.05 was considered significant.

## 3. Results

### 3.1. Age Related Changes in Bone Mass of Spine in Males and Females Derived by QCT

**Figure 1:**
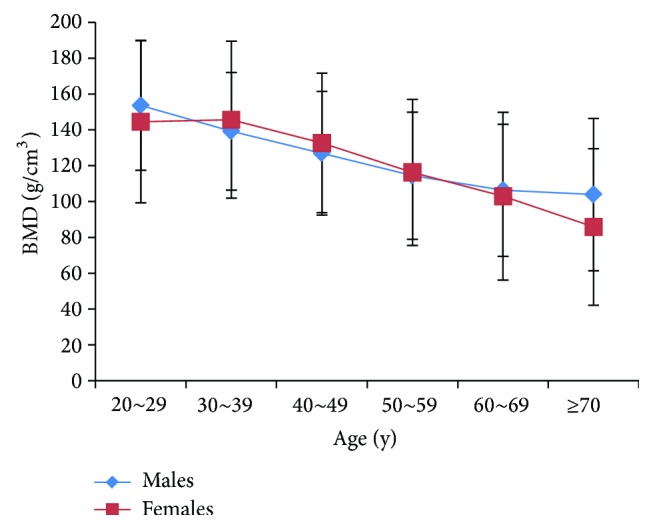
Bone mineral density (in grams per cubic centimeter) changes associated with age for males and females derived by QCT spine.

The peak vBMD values of the lumbar spine was observed at 30 to 39 years in females (145.73 ± 43.78, mg/cm^3^) and 20 to 29 years in males (153.60 ± 36.18, mg/cm^3^). In both sexes, aging was accompanied by a decrease in vBMD after peak bone mass (Figure 1).

### 3.2. The Osteoporosis QCT Detection Rates with vBMD versus aBMD in Males and Females

In males, of the 330 participants, 46 (13.94%) were found to have osteoporosis QCT by QCT Pro volumetric spine BMD measures; the osteoporosis QCT detection number for CTXA Hip area BMD measurements was 9 (2.73%). There was no significant difference in the osteoporosis QCT detection rates using chi-square test (chi-square value = 2.901; *P* = 0.089). However, of the 196 participants in <50 y males, 11 (5.6%) were found to have osteoporosis QCT by vBMD; 4 (2.0%) were detected to have osteoporosis QCT by aBMD. Roughly 3.6% of them differed in osteoporosis QCT detection rates at the spine and hip by QCT. Moreover, in ≥50 y males, of the 134 participants, 35 (26.1%) were found to have osteoporosis QCT by vBMD; the osteoporosis QCT detection number for CTXA Hip area BMD measurements was 5 (3.7%) (Table 1).

In females, of the 496 participants, 109 (21.98%) were found to have osteoporosis QCT by QCT Pro volumetric spine BMD measures; the osteoporosis QCT detection number for CTXA Hip area BMD measurements was 46 (9.27%). There was a significant difference in the osteoporosis QCT detection rates using chi-square test (chi-square value = 124.86; *P* = 0.000), with QCT spinal vBMD detecting osteoporosis QCT more frequently than hip aBMD did. Further, of the 225 participants in <50 y females, 20 (8.9%) were found to have osteoporosis QCT by vBMD; 2 (0.9%) were detected to have osteoporosis QCT by aBMD. Roughly 8.0% of them differed in osteoporosis QCT detection rates at the spine and hip by QCT. In ≥50 y females, of the 271 participants, 89 (32.8%) were found to have osteoporosis QCT by vBMD; the osteoporosis QCT detection number for CTXA Hip area BMD measurements was 44 (16.2%) (Table 2).

### 3.3. Qualitative Skeletal Status Category Agreement between Spine and Hip by QCT Measurement

**Figure 2:**
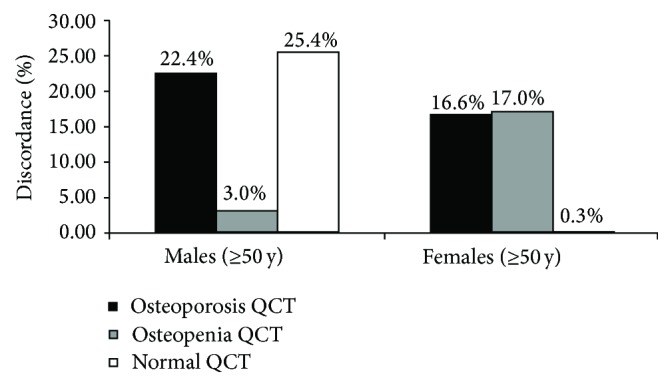
Discordances in skeletal status category between lumbar spine and hip in older males and females.

Osteoporosis QCT, osteopenia QCT, and normal QCT detection rates agreement between QCT spine and CTXA Hip is 3.7%, 32.1%, and 38.8% in older males, respectively, and 16.2%, 29.5%, and 37.3% in older females. Roughly 22.4% and 16.6% discordances was found in osteoporosis QCT detection rates between spine and hip by QCT in older males and females, respectively. 3.0% and 17.0% discordances was found in osteopenia QCT detection rates; 25.4% and 0.3% discordances was found in normal QCT bone mass detection rate (Figure 2).

### 3.4. Results of the Correlation and Kappa Test between QCT vBMD and aBMD

In both males and females, all BMD variables were nonnormally distributed variables. QCT vBMD was positively correlated with aBMD (*r* = 0.130, *P* < 0.05) in males; the correlations between QCT vBMD and aBMD were strong positively correlated in females (*r* = 0.662, *P* < 0.01). In further consistency analysis, in <50 y males, Kappa coefficient = 0.113 and *P* = 0.072; in ≥50 y males, Kappa coefficient = 0.110 and *P* = 0.056; in <50 y females, Kappa coefficient = 0.202 and *P* = 0.000; in ≥50 y females, Kappa coefficient = 0.360 and *P* = 0.000.

## 4. Discussion

QCT could provide the similar results as conventional DXA [10], which may be useful in evaluation of bone mass. It has been generally accepted that peak bone mass at any skeletal site is attained in both sexes during the midthirties. Bone mass decreases significantly with aging both in middle-aged and elderly men and women [11, 12]. In present study, the peak vBMD values of the spine were observed at 30 to 39 years in Chinese women and at 20 to 29 years in Chinese men. Aging was accompanied by a decrease in vBMD after peak bone mass in both sexes.

BMD measurement for DXA has been used as the gold standard in the clinical diagnosis of osteoporosis. QCT has a number of advantages over DXA in BMD measurement [13]. QCT is able to analyze not only vBMD of trabecular and cortical bone compartment separately, but also geometry and biomechanical parameters in bone such as cross-sectional area, cortical bone thickness, section modulus, and buckling ratio. The analysis of geometry and biomechanical parameters at hip could provide better prediction of hip fracture risk [14]. Despite these, one study showed a significant difference in osteoporosis detection rates between DXA and QCT, providing clinical evidence that QCT has a greater diagnostic sensitivity than DXA [15].

As we know, changes of BMD varied according to skeletal site. The sites of BMD most commonly measured are the lumbar spine and hip. Osteoporotic bone loss occurs mainly in trabecular bone. Many clinical guidelines also recommend lumbar spine measurements to assess skeletal status [16, 17]. In general, QCT is most applied in the lumbar spine to measure trabecular BMD. For the BMD of spinal trabecular bone, thresholds of 80 mg/cm^3^ were suggested for osteoporosis. QCT data indicated the detection rate was 46.4% for spinal trabecular BMD in postmenopausal women [15]. In our study, the osteoporosis detection rates for QCT vBMD were 32.8% and 26.1% in ≥50 y women and men, respectively. The lower detection rate of lumbar spine osteoporosis QCT may be due to an unclear distinction between menopausal and postmenopausal status in women.

In addition to the lumbar spine, CTXA for BMD measurement at the hip has been proposed and developed for research [18]. CTXA Hip BMD estimates provides the same clinical utility as that afforded by DXA although the radiation exposure is about 50 times as high [8, 9]. Previous study demonstrated that the precision of CTXA duplicate hip scans was slightly better than DXA [8]. Cheng et al. [19] suggested the CTXA aBMD and *T*-score can be used in the diagnosis and management of osteoporosis as a substitute of DXA aBMD. Based on DXA BMD measurements, in 2005-2006, 49% of older US women had osteopenia and 10% had osteoporosis at the femur neck. In men, 30% had femur neck osteopenia and 2% had femur neck osteoporosis [20]. Based on CTXA aBMD measurements, our study showed 46.5% of older Chinese women had osteopenia and 16.2% had osteoporosis at hip. In older men, 32.1% had hip osteopenia and 3.7% had hip osteoporosis for CTXA BMD. Compared to DXA BMD measurements, the similar osteopenia and the increased osteoporosis detection rate were found by CTXA aBMD measurement in both men and women.

Our study showed QCT vBMD was positively correlated with aBMD both in males and females; nevertheless in further Kappa consistency analysis, Kappa value was less than 0.4 in both men and women; that implied agreement was not found in two diagnostic measures. This study showed the osteoporosis QCT detection rates for CTXA Hip aBMD measures were 9.27% and 2.73% in women and men, respectively. However, those were 21.98% and 13.94% for QCT Pro volumetric spine BMD. Furthermore depending on age, in ≥50 years women and men, the osteoporosis QCT detection rates for CTXA Hip aBMD measures were 16.2% and 3.7%, respectively, and for QCT Pro spine vBMD were 32.8% and 26.1%. There was a significant difference in osteoporosis QCT detection rates between two measurements providing clinical evidence that QCT spine vBMD has a greater diagnostic sensitivity than hip aBMD. This may be due to site-specific differences; trabecular bone can have an advantage of superior sensitivity due to the higher metabolic rate of turnover [9]. Many studies have reported greater discordance in osteoporosis diagnoses between skeletal sites by DXA measurement [21–24]. Recent study indicated that at least half of patients tested by DXA will demonstrate *T*-score discordance between spine and total hip measurement sites; discordance BMD was lower in lumbar spine than total hips [25]. In this study, osteoporosis QCT detection rates discordance between spine and hip measurement sites is 22.4% for males and 16.6% for females. It implied that one site measurement by QCT could be misclassified as not osteoporotic.

Our study has several limitations. There were no BMD measurements of the lumbar spine and hip by DXA in the meantime; there was no clear distinction between menopausal and postmenopausal status in women.

## 5. Conclusions

In summary, age related changes in bone mass derived by QCT measurements in eastern Chinese were determined. Qualitative skeletal status category was available for reference by QCT BMD of the spine and hip in Chinese adults. QCT vBMD were positively correlated with aBMD. However, poor consistency results were detected in the osteoporosis diagnosis between CTXA Hip aBMD measurements and QCT Pro spine vBMD measurements. Compared to CTXA Hip aBMD, QCT spine vBMD may be more sensitive for detecting osteoporosis QCT.

## Figures and Tables

**Table 1:** The diagnostic rates classification with vBMD versus aBMD in males.

Location	Osteoporosis QCT (%)	Osteopenia QCT (%)	Normal QCT (%)
Age <50 y (*n* = 196)			
Lumbar spine (vBMD)	11 (5.6%)	47 (24.0%)	138 (70.4%)
Hip (aBMD)	4 (2.0%)	54 (27.6%)	138 (70.4%)
Age ≥50 y (*n* = 134)			
Lumbar spine (vBMD)	35 (26.1%)	47 (35.1%)	52 (38.8%)
Hip (aBMD)	5 (3.7%)	43 (32.1%)	86 (64.2%)

**Table 2:** The diagnostic rates classification with vBMD versus aBMD in females.

Location	Osteoporosis QCT (%)	Osteopenia QCT (%)	Normal QCT (%)
Age <50 y (*n* = 225)			
Lumbar spine (vBMD)	20 (8.9%)	50 (22.2%)	155 (68.9%)
Hip (aBMD)	2 (0.9%)	57 (25.3%)	166 (73.8%)
Age ≥50 y (*n* = 271)			
Lumbar spine (vBMD)	89 (32.8%)	80 (29.5%)	102 (37.6%)
Hip (aBMD)	44 (16.2%)	126 (46.5%)	101 (37.3%)

## References

[1] Lochmüller E.-M., Bürklein D., Kuhn V. (2002). Mechanical strength of the thoracolumbar spine in the elderly: prediction from in situ dual-energy X-ray absorptiometry, quantitative computed tomography (QCT), upper and lower limb peripheral QCT, and quantitative ultrasound. *Bone*.

[2] Grampp S., Jergas M., Glüer C. C., Lang P., Brastow P., Genant H. K. (1993). Radiologic diagnosis of osteoporosis: current methods and perspectives. *Radiologic Clinics of North America*.

[3] Ott S. M. (1991). Methods of determining bone mass. *Journal of Bone and Mineral Research*.

[4] Engelke K., Adams J. E., Armbrecht G. (2008). Clinical use of quantitative computed tomography and peripheral quantitative computed tomography in the management of osteoporosis in adults: the 2007 ISCD Official Positions. *Journal of Clinical Densitometry*.

[5] Kanis J. A., McCloskey E. V., Johansson H., Oden A., Melton L. J., Khaltaev N. (2008). A reference standard for the description of osteoporosis. *Bone*.

[6] Faulkner K. G., von Stetten E., Miller P. (1999). Discordance in patient classification using T-scores. *Journal of Clinical Densitometry*.

[7] American College of Radiology (2008). *ACR-SPR-SSR Practice Parameter for the Performance of Quantitative Computed Tomography (QCT) Bone Densitometry (Amended 2014 Resolution 39)*.

[8] Pickhardt P., Bodeen G., Brett A., Brown J. K., Binkley N. (2014). Comparison of femoral neck BMD evaluation obtained using lunar DXA and QCT with asynchronous calibration from CT colonography. *Journal of Clinical Densitometry*.

[9] Khoo B. C. C., Brown K., Cann C. (2009). Comparison of QCT-derived and DXA-derived areal bone mineral density and T scores. *Osteoporosis International*.

[10] Cheng Q., Zhu Y. X., Zhang M. X., Li L. H., Du P. Y., Zhu M. H. (2012). Age and sex effects on the association between body composition and bone mineral density in healthy Chinese men and women. *Menopause*.

[11] Iwamoto J., Takeda T., Ichimura S., Tsukimura Y., Toyama Y. (2000). Age-related changes in cortical bone in men: Metacarpal Bone Mass Measurement Study. *Journal of Orthopaedic Science*.

[12] Iwamoto J., Takeda T., Otani T., Yabe Y. (1998). Age-related changes in cortical bone in women: Metacarpal Bone Mass Measurement Study. *Journal of Orthopaedic Science*.

[13] Adams J. E. (2009). Quantitative computed tomography. *European Journal of Radiology*.

[14] Nonaka K., Uchiyama S. (2011). Assessment of volumetric bone mineral density and geometry for hip with clinical CT device. *Clinical Calcium*.

[15] Li N., Li X. M., Xu L. (2013). Comparison of QCT and DXA: osteoporosis detection rates in postmenopausal women. *International Journal of Endocrinology*.

[16] Baim S., Binkley N., Bilezikian J. P. (2008). Official positions of the international society for clinical densitometry and executive summary of the 2007 ISCD position development conference. *Journal of Clinical Densitometry*.

[17] National Osteoporosis Foundation (2008). *Clinician's Guide to Prevention and Treatment of Osteoporosis*.

[18] Cann C. E., Adams J. E., Brown J. K., Brett A. D. (2014). CTXA hip—an extension of classical DXA measurements using quantitative CT. *PLoS ONE*.

[19] Cheng X. G., Wang L., Wang Q. Q., Ma Y. M., Su Y. B., Li K. (2014). Validation of quantitative computed tomography-derived areal bone mineral density with dual energy X-ray absorptiometry in an elderly Chinese population. *Chinese Medical Journal*.

[20] Looker A. C., Melton L. J., Harris T. B., Shepherd J. A. (2010). Prevalence and trends in low femur bone density among older US adults: NHANES 2005-2006 compared with NHANES III. *Journal of Bone and Mineral Research*.

[21] Fink H. A., Harrison S. L., Taylor B. C. (2008). Differences in site-specific fracture risk among older women with discordant results for osteoporosis at hip and spine: study of osteoporotic fractures. *Journal of Clinical Densitometry*.

[22] Stoch S. A., Wysong E., Connolly C., Parker R. A., Greenspan S. L. (2000). Classification of osteoporosis and osteopenia in men is dependent on site-specific analysis. *Journal of Clinical Densitometry*.

[23] Leslie W. D., Tsang J. F., Caetano P. A., Lix L. M., Manitoba Bone Density Program (2007). Number of osteoporotic sites and fracture risk assessment: a cohort study from the manitoba bone density program. *Journal of Bone and Mineral Research*.

[24] O'Gradaigh D., Debiram I., Love S., Richards H. K., Compston J. E. (2003). A prospective study of discordance in diagnosis of osteoporosis using spine and proximal femur bone densitometry. *Osteoporosis International*.

[25] Younes M., Ben Hammouda S., Jguirim M. (2014). Discordance between spine and hip Bone Mineral Density measurement using DXA in osteoporosis diagnosis: prevalence and risk factors. *La Tunisie Médicale*.

